# Effect of the desolventizing/toasting process on chemical composition and protein quality of rapeseed meal

**DOI:** 10.1186/s40104-016-0095-7

**Published:** 2016-06-18

**Authors:** Rainer Mosenthin, Ulrike Messerschmidt, Nadja Sauer, Patrick Carré, Alain Quinsac, Friedrich Schöne

**Affiliations:** University of Hohenheim, Institute of Animal Science, Emil-Wolff-Strasse 10, Stuttgart, 70599 Germany; Present address: KWS SAAT SE, Grimsehlstrasse 31, Einbeck, 37555 Germany; Present address: Landwirtschaftliche Untersuchungs- und Forschungsanstalt Speyer, Obere Langgasse 40, Speyer, 67346 Germany; OLEAD, 11 rue Monge, Parc Industriel, Pessac, 33600 France; Terres Inovia, 11 rue Monge, Parc Industriel, Pessac, 33600 France; Thuringian State Institute of Agriculture, Naumburger Strasse 98, Jena, 07743 Germany

**Keywords:** Anti-nutritional factor, Glucosinolate, Processing, Protein quality, Rapeseed meal

## Abstract

**Background:**

During processing in a desolventizer/toaster (DT), rapeseed meal (RSM) is heated to evaporate the hexane and to reduce the level of heat-labile anti-nutritional factors such as glucosinolates (GSL). However, excessive heat treatment may reduce amino acid (AA) content in addition to lower AA digestibility and availability in RSM. The objective of the present study was to produce from one batch of a 00-rapeseed variety (17 μmol GSL/g dry matter (DM), seed grade quality) five differently processed RSM under standardized and defined conditions in a pilot plant, and to determine the impact of these different treatments on protein solubility and chemical composition, in particular with regard to contents of AA including reactive Lys (rLys) and levels of total and individual GSL.

**Methods:**

Four RSM were exposed to wet toasting conditions (WetTC) with increasing residence time in the DT of 48, 64, 76, and 93 min. A blend of these four RSM was further processed, starting with saturated steam processing (< 100 °C) and followed by exposure to dry toasting conditions (DryTC) to further reduce the GSL content in this RSM.

**Results:**

The contents of neutral detergent fiber and neutral detergent fiber bound crude protein (CP) increased linearly (*P* < 0.05), as residence time of RSM in the DT increased from 48 to 93 min, whereas contents of total and most individual GSL and those of Lys, rLys, Cys, and the calculated ratio of Lys:CP and rLys:CP decreased linearly (*P* ≤ 0.05). The combination of wet heating and DryTC resulted in the lowest GSL content compared to RSM produced under WetTC, but was associated with lowest protein solubility.

**Conclusions:**

It can be concluded that by increasing residence time in the DT or using alternative processing conditions such as wet heating combined with DryTC, contents of total and individual GSL in RSM can be substantially reduced. Further *in vivo* studies are warranted to elucidate if and to which extent the observed differences in protein quality and GSL content between RSM may affect digestibility and bioavailability of AA in monogastric animals.

## Background

Rapeseed meal (RSM) is a by-product of oil processing and commonly used as a protein source for livestock. Among protein feedstuffs, production of RSM ranks in second place behind soybean meal [1, 2]. Despite its well-balanced amino acid (AA) profile [3], in particular its relatively high content of sulfur AA in comparison to other protein feedstuffs including soybean meal [4, 5], the use of RSM in diets for monogastric animals is often limited due to the presence of several anti-nutritional factors. These include low digestible and indigestible fiber fractions such as neutral detergent fiber (NDF), acid detergent fiber (ADF), acid detergent lignin (ADL; [6, 7]) and different glucosinolates (GSL; [8–10]). Depending on the type and level of GSL in the diet of monogastric animals, negative effects on feed intake and growth performance [8, 11–14], in addition to liver- and thyroid-hypertrophy [15] have been reported.

The transition from so called 0-rapeseed (RS) varieties, characterized by low erucic acid content (< 20 g/kg of total fatty acid content; [16]) but high total GSL content of 50–100 μmol/g seed [11, 15], to 00-RS varieties resulted in considerably lower contents of GSL (< 25 μmol total GSL/g seed; [17]). Consequently, the use of RS products in livestock feeding increased worldwide [16, 18]. For example, the production of RSM in Germany went up from less than two million tons in 2000 to more than four million tons in 2011 [19]. With the introduction of 00-RS varieties, the content of total GSL in solvent extracted and toasted RSM decreased from 150 μmol total GSL/g dry matter (DM) in 0-RSM to levels ranging between 1–22 μmol total GSL/g DM in 00-RSM [20]. Nowadays, the content of total GSL in RSM produced in Germany from commercially available 00-RS varieties averages 8.8 μmol total GSL/g RSM [21].

As GSL are known to be heat-labile, thermal treatment during processing of RS, especially during the desolventizing/toasting process, reduces their contents in RSM substantially [3]. Thermal processing of oilseed meals in the desolventizer/toaster (DT) is primarily used to remove the hexane needed for oil extraction. Temperature, steam pressure, and duration of heat treatment during processing are considered to be the main factors responsible for reduction of GSL in RSM [22]. Excessive heat treatment, however, may result in the degradation of AA, thus reducing protein quality of RSM [23]. Therefore, there is a need for optimizing the processing conditions during the desolventizing/toasting process by maximizing removal of GSL in RSM, but without reducing protein solubility, AA contents, and AA digestibility and bioavailability [3, 24, 25].

There are several reports describing the effect of heat treatment, in part under wet conditions, on the degradation of GSL in RSM (*e.g.* [3, 4, 26]). However, no study has been published so far, in which the combined effect of different processing conditions (*e.g.* steam pressure, temperature, residence time in the DT) during manufacturing of RSM on their chemical composition has been assessed. The objective of the present study was to produce in a pilot plant under standardized and defined conditions from one batch of a 00-RS variety five differently processed RSM, and to determine the impact of these different treatments on protein solubility and chemical composition of these RSM including contents of AA and reactive Lys (rLys), and levels of total and individual GSL.

## Methods

### Rapeseed selection

For the production of RSM, the non-genetically modified winter type RS variety Lorenz (*Brassica napus* L., seed grade quality) was used, supplied by Norddeutsche Pflanzenzucht (Hans-Georg Lembke KG, Hohenlieth, Germany). According to breeder’s information, the erucic acid content was below 10 g/kg of total fatty acid content. The total GSL content of this RS variety was 17 μmol/g DM, which is in accordance with the present European Union quality standard for RS [17], and corresponds to average GSL contents in RS varieties grown in the European Union in 2010 [27]. In total, six big bags containing 1,000 kg of RS each were shipped to the pilot plant OLEAD (Pessac, France) to manufacture under standardized and defined conditions five differently processed RSM.

### Rapeseed meal processing

**Fig. 1:**
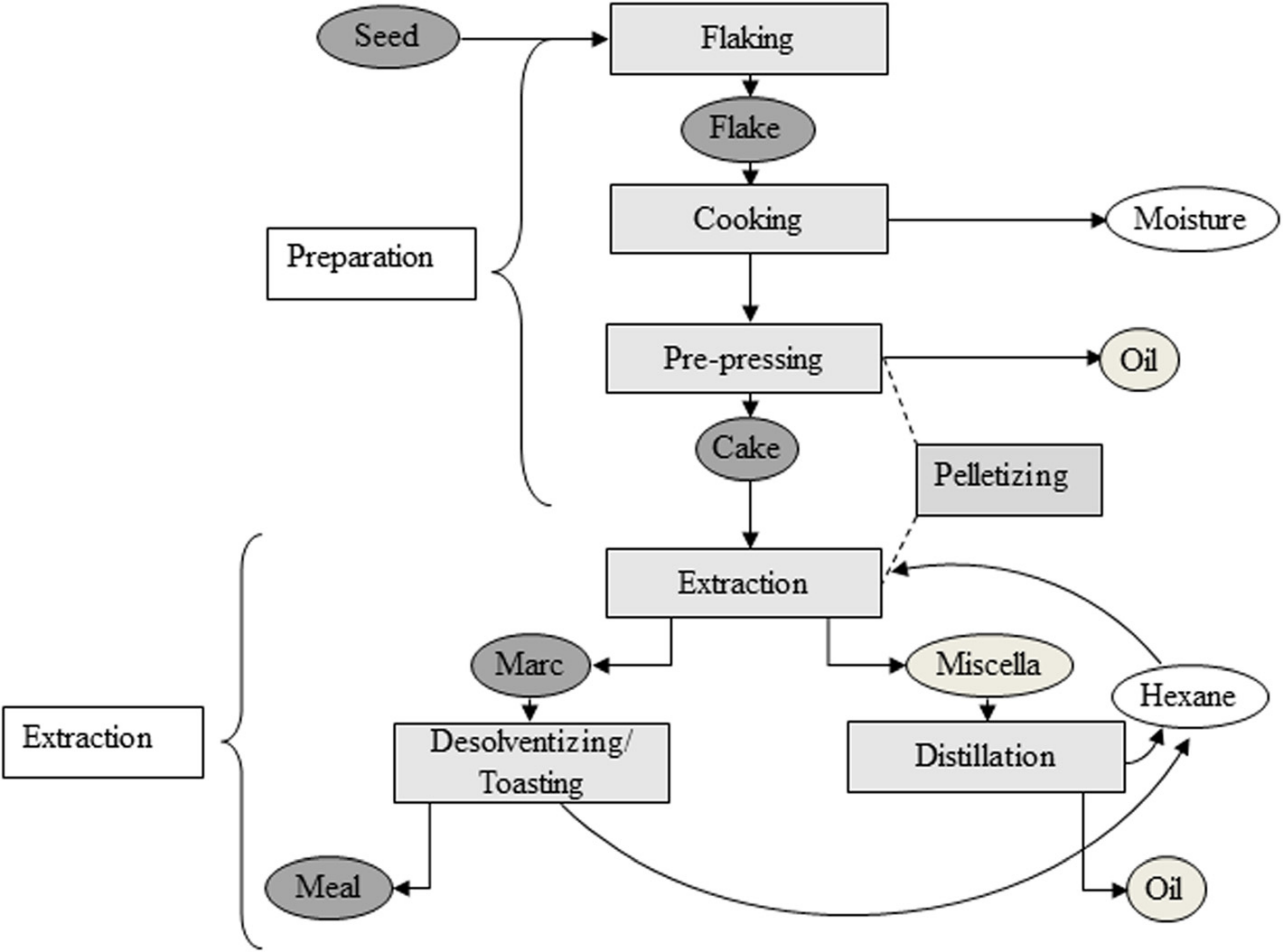
Flow chart of rapeseed processing in a pilot plant (OLEAD, Pessac, France)

Rapeseed meal processing usually includes cleaning, flaking, cooking, pressing, solvent extraction, and desolventizing/toasting [28]. However, in the present study, RS of seed quality was used, thus the cleaning process became redundant. In total, five differently processed RSM were produced from two rapeseed cakes (RSC), referred to as RSC I and RSC II. These RSC were manufactured from the same RS batch after flaking, cooking, and mechanical pressing of the RS. In Fig. 1, the flow chart of the processing steps used in this study is presented.

#### Flaking

Flaking of RS is applied to rupture the seed coat and flattening the cotyledons, thus enhancing the oil output during processing [29]. It is recommended that optimal thickness of the flakes for maximum oil yield should range between 0.30 and 0.38 mm [28].

In the present study, flaking of the raw RS resulted in a thickness of 0.30 mm by passing the RS through two contra-rotating smooth cylinders (Damman Croes, Roeselare, Belgium) of 500 mm diameter each. The space between the cylinders was adjusted to 0.30 mm by hydraulic jacks.

#### Cooking

The RS Flakes are cooked to reduce the moisture content of the flake and heating the material, thus reducing oil viscosity and thereby promoting coalescence of oil droplets, which increases the diffusion rate of solvent into the RSC during the solvent extraction process [30]. Cooking also contributes to the inactivation of enzymes such as myrosinase and lipase [3]. Myrosinase, for example, is involved in the hydrolysis of GSL, which results in the formation of differently volatile degradation products including isothiocyanates, nitriles, thiocyanates, or oxazolidinethiones [3, 14].

In the present study, the RS flakes were cooked in a horizontal cooker (La Mécanique Moderne, Arras, France). This cooker was made up of two superposed horizontal cylinders (cooker and dryer), each of 900 mm in diameter and 2,000 mm in length. The walls of these cylinders were heated by circulating mineral oil to use heat transmission for adjusting the temperature of the RS flakes in the cooker. The convection of heat through the content of the cooker was forced by continuous stirring of helical ribbon. Feeding of the upper cylinder, referred to as cooker, was provided by a volumetric feeder fitted with an anti-bridging agitator. The discharge was operated by sliding gates, located on the extremity of the cylinders at half height. These gates were commanded by a detector located in the hopper of the discharging screw. As soon as the detector was covered, the gates were closed and reciprocally they were opened when the material in the hopper had disappeared. In the present study, the residence time of the flakes in the cooker for producing the two RSC was on average 68 min, and the temperature in the dryer (lower heating device) averaged 94 °C. This second cylinder was connected to a fan to eliminate the moisture from the drying material. The moisture content of the RS flakes after cooking averaged 48 g/kg for production of RSC I and RSC II.

#### Pressing

After cooking, RS flakes were pre-pressed in a large screw press to remove about 60 to 70 % of their oil content [31, 32]. This mechanical extraction was done by compression of the oil bearing material due to a progressive reduction of the volume enclosing this material during its forward displacement. The reduction of volume results from the tapered design and a reduction of the worm pitch, but compression is not linear. The worm sections are separated by conical rings whose function is to increase the resistance force. Crossing these restrictions generates significant pressure phases followed by relaxation areas. The screw is enclosed in a cage formed by longitudinal bars, held by a heavy frame. The bars are separated by metallic spacers to allow the oil to flow between the bars while the RSC was discharged in handling trolleys of 150 L for transportation to the extraction plant. This procedure results in the production of RSC suitable for the subsequent solvent extraction [28].

The pilot plant is equipped with a screw press (MBU 75 model, La Mécanique Moderne, Arras, France), which is characterized by a cage diameter of 180 mm in the feeding area and 150 mm in the compressing area. Total length of the screw is 2,000 mm, from which one third is located in the feeding area. The worm assembly has seven worm sections and seven smooth sections. The screw rotation speed was set to 21.2 and 15.6 rpm for the production of RSC I and RSC II, respectively. The average temperature within the press was 77 °C and the throughput averaged 371 kg/h in both runs. Between 60 and 68 % of the seed’s oil content was removed after pressing of the flakes was completed. Due to pressure, the tiny flakes were compressed into large RSC fragments. After pressing, RSC fragments were pelletized in a pellet mill CLM 200 (La Meccanica s.r.l. di Reffo, Cittadella, Italy) by means of a die with perforations of 90 mm in length and 5 mm in diameter. Pelletizing the RSC at very low pressure aims at reducing and homogenizing RSC fragments different in size, thus this procedure is not comparable with pelleting in compound feed production. Due to increased macroporosity of the pellets, efficient percolation of the solvent during the extraction process is achieved.

#### Solvent extraction process

The second step of oil removal takes places in a solvent extractor to remove most of the residual oil in RSC with hexane as solvent. A continuous belt extractor moves the RSC and the miscella (hexane plus oil) in opposite directions to achieve a continuous counter current extraction [33]. Series of pumps move the miscella over the RSC, so that the miscella with the highest concentration in oil is used to extract the entering RSC with the highest concentration in oil and, at the opposite extremity of the extractor, pure hexane washes the lowest concentrated RSC [28]. According to the extractor design, solvent percolates through the RSC or submerges it, allowing the diffusion of the lipids into the liquid phase. After leaving the solvent extractor, the resulting end product, referred to as marc (solvent saturated RSC), has less than 10 g oil/kg DM [28]. The residual miscella contains generally 250–300 g/kg oil and 700–750 g/kg hexane. The hexane is removed by distillation from the oil and recycled [34].

**Fig. 2:**
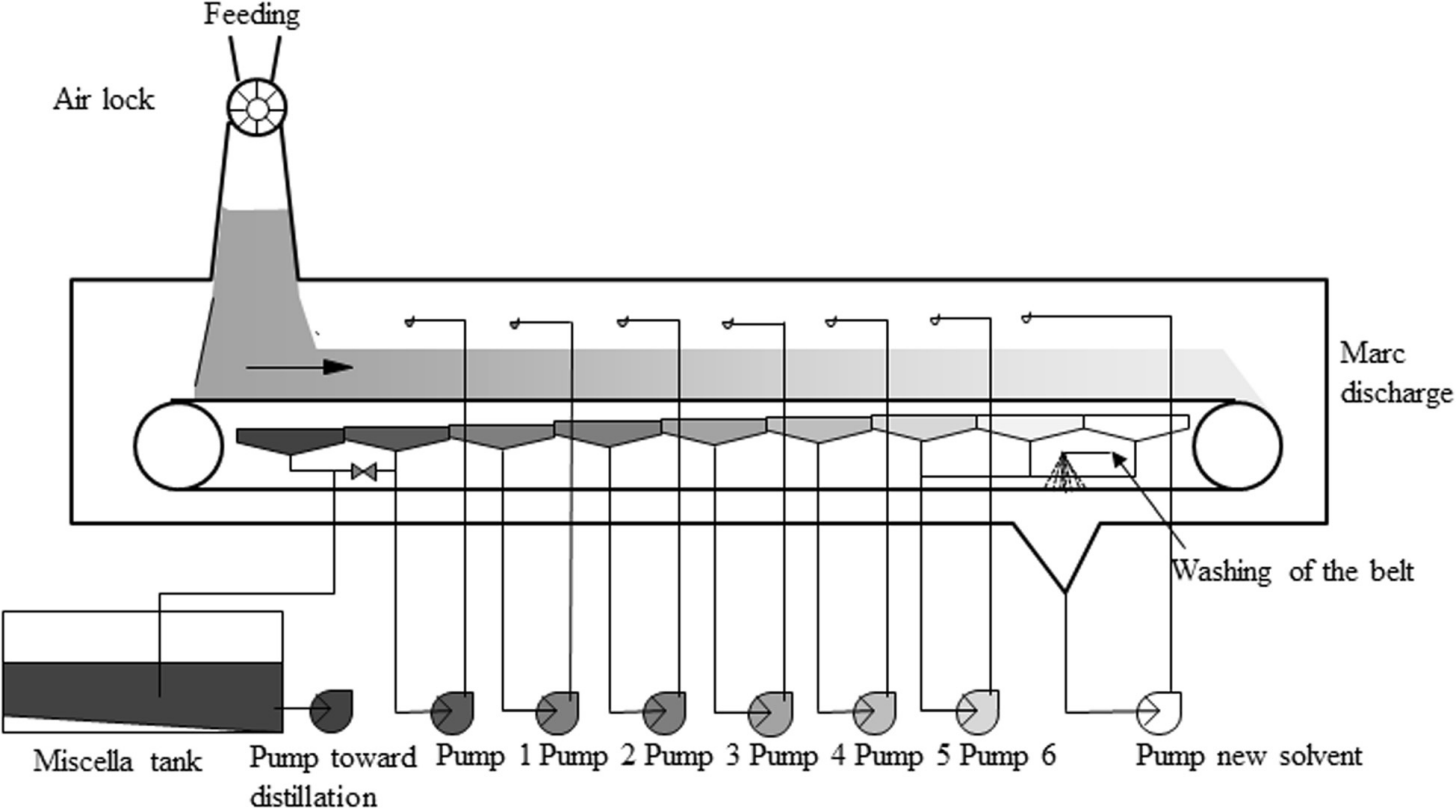
Scheme of continuous belt extractor (Desmet Ballestra, Zaventem, Belgium)

The continuous belt extractor (Desmet Ballestra, Zaventem, Belgium) used in the present study was equipped with a belt of 0.4 m in width and 4.0 m in length, and had a capacity of bearing a layer of 0.4 m of solid material. The counter current flow extraction was carried out by six loops of miscella recirculation (Fig. 2) and the addition of 250 L hexane/h. To maximize oil extraction, the temperature of the miscella was set between 50 °C and 55 °C. The speed of the belt was adjusted to a feeding rate of 150 kg RSC/h. After solvent extraction, the marcs of RSC I and RSC II had an average temperature of 50 °C, and were transferred to the DT.

#### Desolventizing/toasting process

Desolventization in the DT aims at removing all the solvent held in the marc by means of evaporation and steam stripping. The DT is a closed vessel consisting of a vertical stack of cylindrical gas-tight pans, which are steam heated from the base. Starting from the top of the DT, the marc was spread on tray one where it was equally distributed by means of a sweep arm [34]. From there, the marc moved continuously from tray to tray through tray openings. The top trays are referred to as pre-desolventizing trays, because the surface of these hot trays produces indirect heat, which is used to separate the evaporating hexane from the flakes without adding moisture [35]. The main trays are used for toasting the product. They are designed to provide both indirect heating and direct steam contact to remove the bulk of the solvent from the marc, and to add a steam in the gaseous phase of the DT which is needed to displace the equilibriums of phases of hexane by reducing the partial pressure of hexane vapors [36]. The combination of temperature and moisture is mandatory to accomplish GSL degradation [25]. Each of the toasting trays has hollow punches for venting vapors from one tray to the next [35]. These vapors ascend counter current to the direction of the marc, and on the very top of the DT an opening is installed for driving the vapors toward a condensor where both hexane and steam are changing their physical state allowing the pressure to remain close to atmospheric pressure [34]. Marc levels on the last three trays are controlled by chutes to ensure a defined throughput [35]. Direct steam is sparged in the DT by the bottom tray [35].

**Fig. 3:**
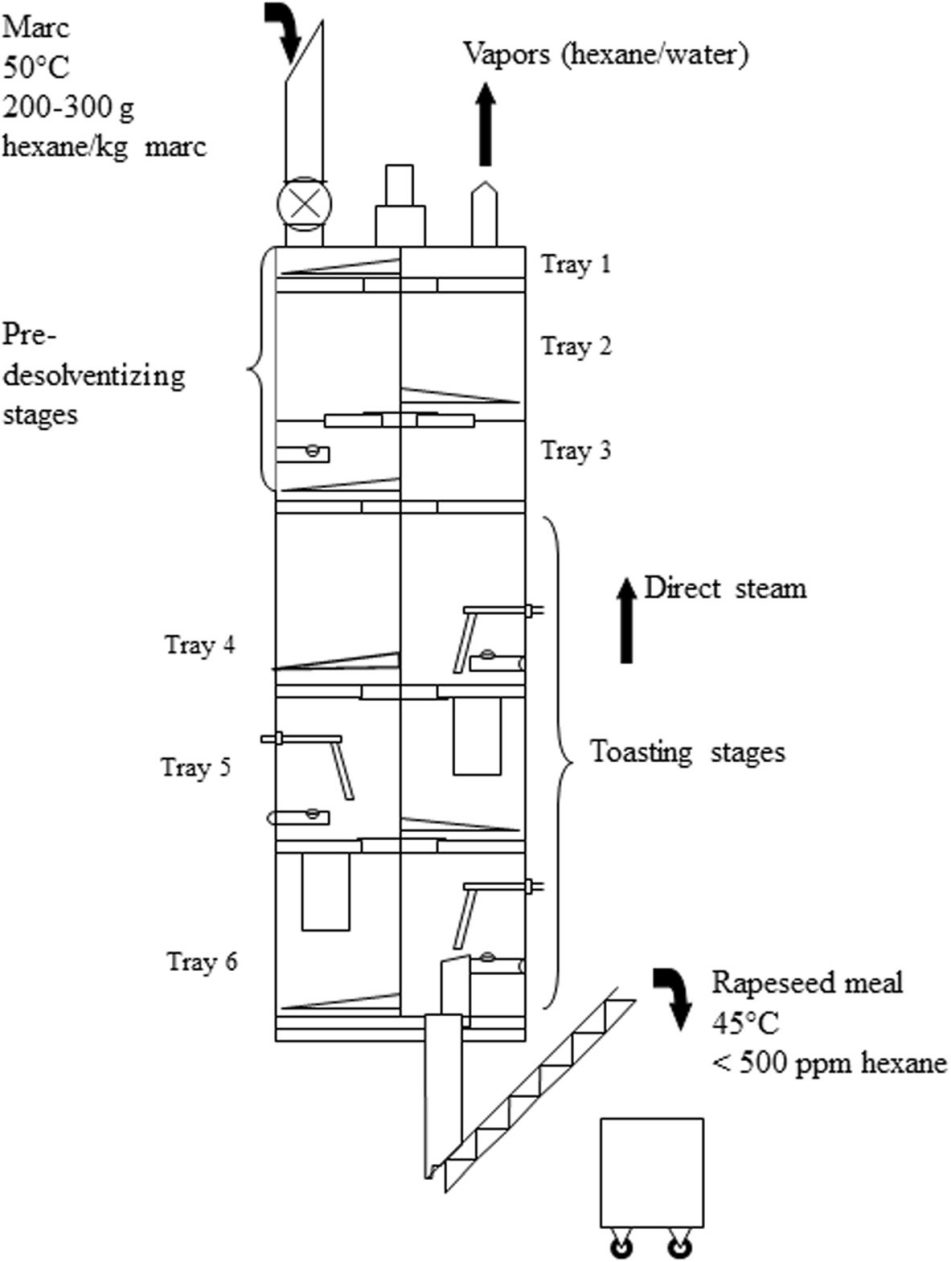
Scheme of desolventizer/toaster (Schumacher type, Desmet Ballestra, Zaventem, Belgium)

In the present study, the DT (Desmet Ballestra, Zaventem, Belgium) was equipped with six trays (Fig. 3). The three upper ones were used as pre-desolventizer, and the three lower ones as toaster. The bottom trays had a diameter of 900 mm; the rotation of sweeps was set at 15 rpm. The desolventizing/toasting conditions in the DT used to manufacture five RSM different in GSL content are summarized in Table 1. Four RSM (A-D) were produced under so called wet toasting conditions (WetTC) using marc from RSC I. This procedure is characterized by the combined application of indirect heat at 850 kPa and direct unsaturated steam (15 kg/h). Residence time in the DT under WetTC for RSM A-D varied between 48 min (RSM A) and 93 min (RSM D) to manufacture RSM different in content of GSL. With increasing residence time in the DT in combination with the use of unstaturated (over-heated) steam, there is higher risk of changing the physical conditions to over-heated steam at temperatures above 100 °C. This results in evaporation of water bound to marc, which, in turn, may create over-toasting conditions. However, for producing a RSM with lowest GSL content, the processing conditions used for producing RSM A-D needed to be further modified. Therefore, marc from RSC II was processed to manufacture four RSM similar to RSM A-D. These RSM from RSC II were pooled to obtain a homogenous batch of RSM with 9 μmol GSL/g DM. This RSM was manually spread over tray six of the DT, which had been indirectly pre-heated with saturated steam at 150 kPa. Thereafter, in a first step, the RSM was exposed for 30 min up to a maximum temperature of 100 °C to direct saturated steam application (30 kg/h). Under these conditions, a hydrothermal treatment with a high water activity is ensured, as direct steam is saturated at temperatures of about 100 °C, thereby heating RSM under wet conditions to degrade GSL. In a second step, dry toasting conditions (DryTC) in the DT were applied for 30 min with indirect steam pressure set at 450 kPa and at an average temperature of 107 °C, i.e. without using steam injection, to further reduce GSL content in this RSM. In comparison to RSM A-D, RSM E received 60 min of additional residence time in the DT.

**Table 1:** Characterization of the desolventizing/toasting processes of rapeseed meals^a^

	Rapeseed meal				
Desolventizing/toasting	A^b^	B^b^	C^b^	D^b^	E^c^
Residence time, min	48	64	76	93	60
Indirect heat, kPa	850	850	850	850	450
Direct steam flow rate, kg/h	15	15	15	15	30
Temperature, °C	126^d^	128^d^	125^d^	115^d^	–
	120^e^	114^e^	129^e^	134^e^	107^e^
Toasting condition	WetTC^f^	WetTC^f^	WetTC^f^	WetTC^f^	DryTC^g^

WetTC = wet toasting conditions; DryTC = dry toasting conditions

^a^Rapeseed meals A-E represent five differently processed rapeseed meals under standardized and defined conditions in a pilot plant

^b^Produced from marc one

^c^Blend of rapeseed meal manufactured from marc two under the same processing conditions as for rapeseed meals A-D

^d^Average temperature on tray five of the desolventizer/toaster. Tray five was not used for production of rapeseed meal E

^e^Average temperature on tray six of the desolventizer/toaster

^f^Use of indirect heat at 850 kPa and direct unsaturated steam (15 kg/h) with mean residence time in the desolventizer/toaster of 48, 64, 76, and 93 min for rapeseed meals A, B, C, and D, respectively

^g^Blend of rapeseed meals produced from marc two was exposed for 30 min up to a maximum temperature of 100 °C to direct saturated steam application (30 kg/h), followed by indirect heat treatment at 107 ° C for 30 min at 450 kPa. Additional residence time in the desolventizer/toaster was 60 min compared to rapeseed meals A-D

The desolventizing/toasting process was completed after the RSM had reached the bottom of the DT. They were chilled by moving them on a screw conveyor, which was cooled with iced water, and then discharged into trolleys. At this time, average temperatures of RSM amounted to approximately 45 °C. Afterwards, all RSM were further cooled down by air ventilation until room temperature was achieved. After the cooling process, the residual hexane content of the RSM, which was determined by means of a gas explosimeter (EX 2000 hand held detector, Oldham, US), was lower than 500 ppm [37]. The RSM were stored in plastic bags before being shipped to the University of Hohenheim (Stuttgart, Germany), where RSM of the same batch were homogenized and stored at −15 °C until further analyses.

### Chemical analyses

Homogeneity of RS among the six big bags used for storage was assessed by means of near infrared reflectance spectroscopy according to method 31.1 [38] prior to processing.

Samples for chemical analyses of RS, RSC, and RSM A-E were ground to pass through a sieve of 0.5 mm mesh screen. Contents of proximate nutrients (except for crude protein (CP)), NDF, ADF, ADL, and sugar were determined according to Naumann and Bassler [38]. Contents of DM were analyzed according to method 3.1, whereas ash and ether extract (EE) contents were measured according to methods 8.1 and 5.1.1 (using petrol ether), respectively. Neutral detergent fiber, expressed as aNDF, was assayed after pre-treatment with a heat stable amylase and expressed inclusive of residual ash (method 6.5.1). Contents of ADF were determined according to method 6.5.2 and expressed inclusive of residual ash. Determination of ADL, expressed as ADL (sa), was performed by solubilization of cellulose with sulphuric acid (method 6.5.3). Sugar content in RSM products was determined by means of ethanol extraction (method 7.1.1). The content of neutral detergent fiber bound crude protein (NDF-CP) was measured according to procedures of Licitra et al. [39]. Protein solubility in potassium hydroxide was determined according to Araba and Dale [40], and was expressed as solubility of total protein soluble in 0.2 % potassium hydroxide. Nitrogen contents in RSC and RSM were analyzed using a gas combustion method according to the official method 990.03 of the AOAC [41], and CP contents in RS products were calculated by multiplying total nitrogen content with the factor 6.25. Ethylenediaminetetraacetic acid was used as a reference standard before and after all nitrogen analyses.

Contents of AA in RSC and RSM were determined by using ion-exchange chromatography with postcolumn derivatization with ninhydrin. The AA were oxidized with performic acid which was neutralized with sodium metabisulfite [42, 43]. Then, they were hydrolyzed by means of 6 mol/L hydrochloric acid for 24 h at 110 °C. Afterwards, AA were quantified with the internal standard method by measuring the absorption of reaction products with ninhydrin at 570 nm. Tryptophan was determined by high-performance liquid chromatography with fluorescence detection (extinction 280 nm, emission 356 nm), after alkaline hydrolysis with barium hydroxide octahydrate for 20 h at 110 °C [44]. Tyrosine was not determined. Contents of rLys in RSC and RSM were determined according to Fontaine et al. [45], which is characterized by the transformation of dietary Lys to its AA analogue homoarginine by means of guanidination with O-methylisourea.

Levels of GSL in RSM were determined by means of reverse phase high-performance liquid chromatography with UV detection, using sinigrin as internal standard [46].

### Statistical analyses

Linear relationships between residence time in the DT and chemical composition, Lys:CP ratio, or rLys:CP ratio in RSM, manufactured under WetTC, were analyzed by the regression (REG) procedure of SAS [47]. The significance level was set at *P* = 0.05.

## Results

### Chemical composition of rapeseed

Near infrared reflectance spectroscopy analysis of the six big bags of RS prior to the processing of RS to RSM verified homogeneity among these batches, as contents of DM, EE, CP, and total GSL ranged from 954 to 958 g/kg, 514 to 522 g/kg DM, 181 to 188 g/kg DM, and 16.7 to 17.1 μmol total GSL/g DM, respectively (data not shown). Due to these low variations in chemical composition between the individual big bags homogeneity was presumed.

Analyzed contents of EE, CP, aNDF, ADF, and ADL (sa) in RS amounted to 506, 185, 258, 214, and 82 g/kg DM, respectively.

### Effect of processing conditions on contents of proximate nutrients, fiber fractions, amino acid composition, and protein quality of rapeseed products

The analyzed chemical composition of RSC I and the five RSM (RSM A-E) is presented in Tables 2 and 3. Residence time in the DT, ranging from 48 to 93 min, showed no effect (*P* > 0.05) on contents of CP, EE, ADF, ADL (sa), ash, and sugar of RSM, which were produced from marc of RSC I. However, there were higher contents of aNDF and NDF-CP as residence time in the DT increased from 48 to 93 min (*P* < 0.05). Moreover, residence time had no effect (*P* > 0.05) on AA composition of RSM, except for contents of Lys, Cys, and rLys, which decreased as residence time in the DT increased (*P* < 0.05) from 48 to 93 min. Accordingly, there was a decrease in Lys:CP ratio (*P* < 0.05) and rLys:CP ratio (*P* = 0.05) with increasing residence time in the DT.

**Table 2:** Chemical composition of rapeseed cake and rapeseed meals (g/kg dry matter, unless otherwise stated)^a^

		Rapeseed meal				
Item	RSC I	A	B	C	D	E
Dry matter, g/kg	943	943	937	941	945	899
Crude protein	294	379	376	380	382	381
Ether extract	210	18	19	19	19	23
aNDF^b^	303	407	417	450	467	476
NDF-CP^b^	–	108	123	154	166	161
ADF	196	246	258	280	280	269
ADL (sa)	86	131	149	152	155	155
Ash	64	81	81	81	81	83
Sugar	–	93	90	86	86	86
Total GSL^c^, μmol/g DM	21	15	12	8	6	4

RSC I = rapeseed cake one; aNDF = neutral detergent fiber with heat stable amylase and expressed inclusive residual ash; NDF-CP = neutral detergent fiber bound crude protein; ADF = acid detergent fiber expressed inclusive residual ash; ADL (sa) = acid detergent lignin determined by solubilization of cellulose with sulphuric acid; GSL = glucosinolates; DM = dry matter

^a^Linear regression analysis refers to the effect of residence time in the desolventizer/toaster on chemical composition of rapeseed meals A-D. Rapeseed meals A-D represent four differently processed rapeseed meals under standardized and defined conditions in a pilot plant (for details see Table 1)

^b^Linear increase (*P* < 0.05)

^c^Linear decrease (*P* < 0.05)

–not determined

**Table 3:** Contents of AA and protein value of rapeseed cake and meals (g/kg dry matter, unless otherwise stated)^a^

		Rapeseed meal				
Item	RSC I	A	B	C	D	E
Indispensable AA						
Arg	17.7	22.1	21.5	20.6	20.6	20.9
His	7.8	10.1	10.0	10.0	10.0	10.0
Ile	11.9	15.3	15.3	15.2	15.0	14.6
Leu	20.9	27.0	26.7	26.8	26.8	26.6
Lys^b^	17.0	19.5	18.8	17.7	17.2	17.2
Met	5.9	7.6	7.4	7.6	7.4	7.4
Phe	11.7	15.3	15.0	15.2	15.1	15.1
Thr	13.4	17.6	17.1	17.3	17.5	17.5
Trp	–	5.1	5.0	5.1	5.1	5.1
Val	15.6	19.7	19.6	19.7	19.1	18.6
Dispensable AA						
Ala	13.2	17.1	16.8	17.0	17.0	16.8
Asp	21.8	28.3	27.7	27.9	27.6	27.4
Cys^b^	7.2	9.1	8.9	8.8	8.7	8.3
Glu	47.0	60.3	59.5	59.9	59.6	59.3
Gly	15.6	20.1	19.8	20.1	20.0	19.7
Pro	17.5	22.4	23.5	24.0	22.8	22.2
Ser	12.5	16.4	16.2	16.0	16.7	16.7
Reactive Lys^b^	13.8	16.0	15.2	13.1	12.5	13.3
Lys:crude protein ratio^b^	0.058	0.051	0.050	0.047	0.045	0.045
Reactive Lys:crude protein ratio	0.047	0.042	0.040	0.034	0.033	0.035
Protein solubility, %	66	42	41	33	32	12

RSC I = rapeseed cake one

^a^Linear regression analysis refers to the effect of residence time in the desolventizer/toaster on amino acid composition and protein quality of rapeseed meals A-D. Rapeseed meals A-D represent four differently processed rapeseed meals under standardized and defined conditions in a pilot plant (for details see Table 1)

^b^Linear decrease (*P* < 0.05)

–not determined

Both contents of proximate nutrients including ADF, ADL (sa), ash, and sugar contents in RSM E were similar to those of RSM A-D, except for EE and aNDF, which were highest in RSM E. Also, AA content of RSM E was similar to that of RSM A-D, except for lower levels of Ile, Val, Asp, Cys and Glu. Moreover, values for rLys, and ratios of rLys:CP and Lys:CP in RSM E were in the range of those obtained for RSM A-D. The protein solubility of RSM A-D showed a numerical decrease from 42 to 32 % (*P* = 0.08) with increasing residence time in the DT from 48 to 93 min under WetTC. However, the processing of RSM E under DryTC resulted in a substantial decrease in protein solubility down to 12 %.

### Effects of processing conditions on glucosinolate contents of rapeseed products

Total and individual GSL contents of RS, RSC I, and RSM A-E, and the degradation rates of GSL are shown in Tables 2 and 4. Total GSL content of RS and RSC I amounted to 17 and 21 μmol/g DM or 34 and 26 μmol/g defatted DM, respectively, whereas total GSL content of the five differently processed RSM ranged from 4 to 15 μmol/g defatted DM. Compared to defatted RS, degradation rate of total GSL in defatted RSM increased with increasing residence time in the DT of 48, 64, 76, and 93 min, amounting to 54, 65, 75, and 83 % (DM basis), respectively. Overall, 76 to 96 % of total indole GSL (4-hydroxyglucobrassicin, glucobrassicin, 4-methoxyglucobrassicin, neoglucobrassicin) and 50 to 88 % of total aliphatic GSL (progoitrin, gluconapoleiferin, gluconapin, glucobrassicanapin) in defatted RSM A-E (DM basis) were degraded during processing compared to defatted RS (DM basis). With increasing residence time in the DT from 48 to 93 min, there was a linear reduction (*P* < 0.05) of most individual GSL in RSM A-D, except for gluconasturtiin, a phenyl GSL, and neoglucobrassicin. Dry toasting conditions further reduced aliphatic and indole GSL contents by 90 and 98 %, respectively, in defatted RSM E in comparison to defatted RS, and neoglucobrassicin had completely disappeared.

**Table 4:** Effect of processing conditions on the contents of total and individual glucosinolates in rapeseed products (μmol/g defatted dry matter, unless otherwise stated)^a^

			Rapeseed meal				
Item	RS	RSC I	A	B	C	D	E
Total glucosinolates^b^	34	26	15	12	8	6	4
Progoitrin^b^	13.4	10.90	6.55	5.01	3.63	2.49	1.72
Gluconapoleiferin^b^	1.41	1.33	0.83	0.61	0.42	0.01	0.17
Gluconapin^b^	7.50	5.88	3.76	2.89	2.12	1.56	0.91
4-Hydroxyglucobrassicin^b^	5.13	3.07	0.98	0.66	0.42	0.24	0.13
Glucobrassicanapin^b^	4.85	4.03	2.55	1.92	1.40	1.01	0.49
Glucobrassicin^b^	0.37	0.34	0.18	0.16	0.11	0.09	0.02
Gluconasturtiin	0.43	0.17	0.18	0.21	0.22	0.17	0.16
4-Methoxyglucobrassicin^b^	0.48	0.63	0.32	0.29	0.16	0.12	0.10
Neoglucobrassicin	0.20	0.08	0.02	0.01	0.01	0.01	n.d.
Degradation rate of total GSL, %^c^		22	54	65	75	83	89
Degradation rate of aliphatic GSL, %^cd^		18	50	62	72	81	88
Degradation rate of indol GSL, %^cd^		33	76	82	89	93	96

RS = rapeseed; RSC I = rapeseed cake one; GSL = glucosinolates; n.d. = not detectable

^a^Linear regression analysis refers to the effect of residence time in the desolventizer/toaster on total and individual glucosinolates contents of rapeseed meals A-D. Rapeseed meals A-D represent four differently processed rapeseed meals under standardized and defined conditions in a pilot plant (for details see Table 1)

^b^Linear decrease (*P* < 0.05)

^c^In comparison to defatted rapeseed

^d^Aliphatic GSL represent the sum of progoitrin, gluconpoleiferin, gluconapin, and glucobrassicanapin; indol GSL that of 4-hydroxyglucobrassicin, glucobrassicin, and neoglucobrassicin

## Discussion

### Effect of processing conditions on contents of proximate nutrients, fiber fractions, amino acid composition, and protein quality of rapeseed products

Generally, manufacturing of RSM results in higher contents of proximate nutrients (except for EE) and AA compared with RSC. Crude protein and EE contents in RSM A-E were in the range of previously reported values [24, 48]. According to Grala et al. [49] and Newkirk et al. [24], desolventizing/toasting during RSM processing had no effect on CP content. This is in agreement with the results of the present study where both, variations in residence time in the DT from 48 to 93 min and in processing conditions (WetTC *vs*. DryTC) did not affect CP contents as well as contents of other proximate nutrients and ADF, ADL (sa), and sugar. However, contents of aNDF and NDF-CP increased, whereas Lys, rLys, Cys contents, and the calculated ratios of Lys:CP and rLys:CP in RSM decreased with increasing residence time in the DT from 48 to 93 min. According to Pastuszewska et al. [50] and Classen et al. [51], the observed increase in aNDF and NDF-CP content due to longer residence time and higher temperature in the DT reflects stronger binding of proteinaceous constituent of CP to fiber fractions inherent in RSM. Moreover, similar to the results of the present study, hydrothermal treatment (WetTC) during desolventizing/toasting may affect protein quality of RSM by reducing their protein solubility, Lys contents, and Lys:CP ratio [25, 50].

Higher contents of NDF and NDF-CP have been attributed to an extended desolventizing/toasting process [50, 51]. The injection of unsaturated steam into the DT during the manufacturing of RSM results in the production of over-heated steam at temperatures above 100 °C, thereby supporting bindings of CP to fiber fractions, especially to NDF and ADF [52, 53]. Accordingly, the highest NDF-CP content was obtained for RSM D, due to longer exposure to unsaturated steam treatment at temperatures above 100 °C.

Elevated contents of NDF-CP and aNDF on the one hand, and lower levels of Lys, rLys, and Lys:CP and rLys:CP ratios on the other hand upon increasing residence time of RSM under WetTC in the DT are considered to be indicators for the so called Maillard reaction [24, 25, 54]. Lysine is the most susceptible AA to Maillard reaction, because it contains an exposed ε-amino group that reacts with reducing sugars [55]. The resulting sugar-AA complex is biologically unavailable for absorption and bound Lys (unreactive) is not utilized [56, 57]. According to Fontaine et al. [45], rLys content is a more sensitive and realistic indicator for the degree of heat damage in feed ingredients than total Lys content. Therefore, it can be assumed that a decrease in rLys content is associated with a reduced digestibility of CP and AA, especially of Lys, Cys, and Arg *in vivo* [42]. Moreover, reduction of Cys contents in RSM due to increasing residence time in the DT from 48 to 93 min may result from the formation of cross-linked compounds that are formed during an advanced Maillard reaction [45, 58, 59]. For RSM E, produced under DryTC, contents of Lys, rLys, and Cys were lower compared to the average of RSM A-D, thus indicating higher heat damage of AA.

According to Pastuszewska et al. [60], lower contents of rLys in RSM are associated with higher NDF-CP levels, but also with a decline in protein solubility. This is in agreement with Jensen et al. [3] who confirmed that a decrease in protein solubility is related to lower Lys content. However, protein solubility was only slightly affected (*P* = 0.08) under these WetTC, whereas under DryTC protein solubility of RSM E was between 20 and 30 %-units lower compared to values for RSM A-D. In conclusion, there is need to assess, if and to which extent the described changes in protein quality among RSM, due to varying processing conditions, correspond to changes in AA digestibility and bioavailability in pigs and poultry.

### Effect of processing conditions on glucosinolate contents of rapeseed meals

It could be shown that with increasing residence time in the DT from 48 to 93 min contents of total and individual GSL in RSM decreased substantially, except for gluconasturtiin and neoglucobrassicin, probably due to low initial levels of these GSL in RS. The observed differences in degradation of individual GSL can be attributed to their varying susceptibility to heat treatment. Under WetTC, increasing residence time in the DT from 48 to 93 min decreased total GSL contents from 54 to 83 % with stronger degradation of indole GSL (76 to 93 %) than aliphatic GSL (50 to 81 %). Further degradation of GSL was observed under DryTC (RSM E), with total, indole, and aliphatic GSL being degraded up to 89, 96, and 88 %, respectively (Table 4). In fact, aliphatic GSL have been proven to be less susceptible to heat treatment than indole GSL [61].

The present findings have been confirmed by Schumann [22] under more practical conditions. In total, 10 different RS were used for production of RSM in 10 different German oil mills. Total GSL contents in the 10 RS ranged from 13 to 16 μmol/g RS. After solvent extraction but before toasting, total GSL contents of these 10 RSM varied between 23 and 27 μmol/g RSM. After toasting, however, total GSL contents among toasted RSM differed considerably, ranging from 4 to 16 μmol/g RSM. This great variation is also reflected in differences in the degradation rate of total GSL upon toasting, varying between 33 and 85 % among the 10 oil mills, with indole GSL being more affected (degradation rate of 70 to 97 %) than aliphatic GSL (degradation rate of 12 to 81 %). According to Schumann [22], intensification of heat treatment during the toasting process is associated with continuous degradation of GSL, thereby resulting in the production of RSM with low GSL contents. The results of the present study and those reported by Schumann [22] have been confirmed by Jensen et al. [3] under laboratory-scale conditions. A batch of RSM was pre-heated at 95 °C for 5 min before being exposed to direct steam application in a “toaster” at 100 °C. With increasing residence time in the toaster of 15, 30, 60, and 120 min, total GSL contents of the RSM decreased from initially 16.2 μmol GSL/g in the pre-heated RSM to 12.3, 8.7, 4.9, and 0.8 μmol/g in the final RSM product which corresponds to degradation rates of 24, 48, 70, and 95 %, respectively. Similar to the results of the present study and those reported by Schumann [22], degradation of indole GSL was higher compared to aliphatic GSL.

Individual GSL and their different degradation products may have varying anti-nutritional effects when included in the diet of monogastric animals [14]. Hydrolysis of GSL depends on the presence of myrosinase (thioglucoside glucohydrolase, EC 3.2.3.1.) which is present in intact cells of cruciferous plants. The hydrolysis products include mainly isothiocyanates, oxazolidinethiones, nitriles, and thiocyanates [8, 9] which are known to affect animals’ health and growth performance negatively. Although no myrosinase mediated hydrolysis of GSL will occur in RSM during and after toasting, some gastrointestinal microorganisms [62] are known to exert myrosinase-like activity [63], thereby hydrolyzing GSL.

## Conclusion

Increasing the residence time under WetTC had detrimental effects on measures of protein quality of the produced RSM such as linear increase in contents of NDF-CP, linear decrease in contents of Lys, rLys, and a lower Lys:CP ratio. A further extension of the residence time in combination with DryTC results in a further reduction of total and individual GSL in RSM*.* The batches of RSM produced under defined and standardized conditions in a pilot plant as described herein will be used in *in vivo* studies with monogastric animals to assess if and to which extent the observed differences in measures of protein quality and GSL reduction between RSM are reflected in changes of digestibility and bioavailability of CP and AA.

## Abbreviations

AA, amino acids; ADF, acid detergent fiber expressed inclusive residual ash; ADL (sa), acid detergent lignin determined by solubilization of cellulose with sulphuric acid; ADL, acid detergent lignin; aNDF, neutral detergent fiber assayed with a heat stable amylase and expressed inclusive residual ash; CP, crude protein; DM, dry matter; DryTC, dry toasting conditions; DT, desolventizer/toaster; EE, ether extract; GSL, glucosinolate; NDF, neutral detergent fiber; NDF-CP, neutral detergent fiber bound crude protein; REG, regression procedure; rLys, reactive Lys; RS, rapeseed; RSC, rapeseed cake; RSM, rapeseed meal; WetTC, wet toasting conditions

## References

[1] FAOSTAT. Food and Agriculture Organization of the United Nations. 2014. http://faostat.fao.org/site/535/default.aspx#ancor. Accessed 2 Mar 2014.

[2] United States Department of Agriculture (USDA). Major Protein Meals: World Supply and Distribution (Commodity View). 2014. http://apps.fas.usda.gov/psdonline/psdReport.aspx?hidReportRetrievalName=Table+02%3a+Major+Protein+Meals%3a+World+Supply+and+Distribution+(Commodity+View)&hidReportRetrievalID=701&hidReportRetrievalTemplateID=5. Accessed 2 Mar 2014.

[3] Jensen SK, Liu Y, Eggum BO (1995). The effect of heat treatment on glucosinolates and nutritional value of rapeseed meal in rats. Anim Feed Sci Technol.

[4] Mansour EH, Dworschák E, Lugasi A, Gaál Ö, Barna É, Gergely A (1993). Effect of processing on the antinutritive factors and nutritive value of rapeseed products. Food Chem.

[5] Woyengo TA, Kiarie E, Nyachoti CM (2010). Energy and amino acid utilization in expeller-extracted canola meal fed to growing pigs. J Anim Sci.

[6] de Lange CFM, Gabert VM, Gillis D, Patience JF (1998). Digestible energy contents and apparent ileal amino acid digestibilities in regular or partial mechanically dehulled canola meal samples fed to growing pigs. Can J Anim Sci.

[7] Kracht W, Dänicke S, Kluge H, Keller K, Matzke W, Hennig U, Schumann W (2004). Effect of dehulling of rapeseed on feed value and nutrient digestibility of rape products in pigs. Arch Anim Nutr.

[8] Bell JM (1984). Nutrients and toxicants in rapeseed meal: A review. J Anim Sci.

[9] Fenwick GR (1984). Rapeseed as an animal feedingstuff - The problems and analysis of glucosinolates. J Assoc Public Anal.

[10] Mailer RJ, McFadden A, Ayton J, Redden B (2008). Anti-nutritional components, fibre, sinapine and glucosinolate content, in Australian canola (*Brassica napus L*.) meal. J Am Oil Chem Soc.

[11] Bell JM (1993). Factors affecting the nutritional value of canola meal: A review. Can J Anim Sci.

[12] Mawson R, Heaney RK, Zdunczyk Z, Kozlowska H (1994). Rapeseed meal-glucosinolates and their antinutritional effects Part 3. Animal growth and performance. Food Nahrung.

[13] Schöne F, Groppel B, Hennig A, Jahreis G, Lange R (1997). Rapeseed meals, methimazole, thiocyanate and iodine affect growth and thyroid. Investigations into glucosinolate tolerance in the pig. J Sci Food Agric.

[14] Tripathi MK, Mishra AS (2007). Glucosinolates in animal nutrition: A review. Anim Feed Sci Technol.

[15] Bourdon D, Aumaître A (1990). Low-glucosinolate rapeseeds and rapeseed meals: Effect of technological treatments on chemical composition, digestible energy content and feeding value for growing pigs. Anim Feed Sci Technol.

[16] Jeroch H, Schöne F, Jankowski J (2008). Composition of rapeseed products and nutritional value for poultry. Arch Geflügelk.

[17] Commission Regulation No. 2316/1999 (1999). Laying down detailed rules for the application of Council Regulation (EC) No. 1251/1999 establishing a support system for producers of certain arable crops. Off J Eur Union.

[18] Quinsac A, Lessire M, Krouti M, Ribaillier D, Coïc JP, Fauduet H, Rollin P (1994). Improvement in the nutritive value of high and low glucosinolate rapeseed meal by aqueous extraction. Anim Feed Sci Technol.

[19] Oil World. 2012. http://www.oilworld.biz/. Accessed 24 Jun 2012.

[20] Dauguet S, Krouti M, Loison JP, Peyronnet C, Quinsac A. A multi-year survey on the chemical composition of rapeseed meal produced in France. In: GCIRC, editors. Proceedings of the 13th International Rapeseed Congress. Prague, Czech Republic; 2011.

[21] Weber M. Rapsextraktionsschrot beweist hohe Qualität – Ergebnisse aus dem deutschlandweiten Monitoring der Fütterungsreferenten 2014. 2015. http://www.proteinmarkt.de/fileadmin/user_upload/Fachartikel_KW_16-Rapsmoitoring_web.pdf. Accessed 26 May 2015.

[22] Schumann W (2005). Untersuchungen zum Glucosinolatgehalt von in Deutschland erzeugten und verarbeiteten Rapssaaten und Rapsfuttermitteln. UFOP-Schriften Heft.

[23] Anderson-Hafermann JC, Zhang Y, Parsons CM (1993). Effects of processing on the nutritional quality of canola meal. Poult Sci.

[24] Newkirk RW, Classen HL, Edney MJ (2003). Effects of prepress-solvent extraction on the nutritional value of canola meal for broiler chickens. Anim Feed Sci Technol.

[25] Newkirk RW, Classen HL, Scott TA, Edney MJ (2003). The digestibility and content of amino acids in toasted and non-toasted canola meals. Can J Anim Sci.

[26] Burel C, Boujard T, Tulli F, Kaushik SJ (2000). Digestibility of extruded peas, extruded lupin, and rapeseed meal in rainbow trout (*Oncorhynchus mykiss*) and turbot (*Psetta maxima*). Aquaculture.

[27] Labalette F, Dauguet S, Merrien A, Peyronnet C, Quinsac A. Glucosinolates content, an important quality parameter monitored at each stage of the French rapeseed production chain. In: GCIRC, editors. Proceedings of the 13th International Rapeseed Congress. Prague, Czech Republic; 2011.

[28] Canola Council of Canada. Canola meal. Feed industry guide, 4th ed., Newkirk R, editor, Canadian International Grains Institute, Winnipeg, Canada. 2009. http://www.canolacouncil.org/media/503589/canola_guide_english_2009_small.pdf. Accessed 2 Mar 2014.

[29] McCurdy SM (1990). Effects of processing on the functional properties of canola/rapeseed protein. J Am Oil Chem Soc.

[30] Ward JA (1984). Pre-pressing of oil from rapeseed and sunflower. J Am Oil Chem Soc.

[31] Bredeson DK (1983). Mechanical oil extraction. J Am Oil Chem Soc.

[32] Vadke VS, Sosulski FW (1988). Mechanics of oil expression from canola. J Am Oil Chem Soc.

[33] Paraíso PR, Cauneto H, Zemp RJ, Andrade CMG (2008). Modeling and simulation of the soybean oil meal desolventizing–toasting process. J Food Eng.

[34] Kemper TG, Shahidi F (2005). Oil Extraction. Bailey's Industrial Oil and Fat Products.

[35] Crown Iron Works Company, A CPM Company. Desolventizer-Toaster. 2010. http://www.crowniron.com/assets/library/brochures/USA/extraction/Crown_DT_Brochure.pdf. Accessed 4 Jun 2016.

[36] Schumacher HO. Apparatus for desolventizing and drying solvent-containing residue meal. Patent No. US4622760; 1986. http://www.google.com/patents/US4622760. Accessed 4 Jun 2016.

[37] Laisney J. L’Huilerie Moderne: Art et technique, Compagnie francaise pour le développement des fibres textiles, Paris, France; 1984.

[38] Naumann C, Bassler R (1976). Verband Deutscher Landwirtschaftlicher Untersuchungs- und Forschungsanstalten.: Methodenbuch Band III. Die Chemische Untersuchung von Futtermitteln. Mit Ergänzungslieferungen 1983, 1988, 1993, 1997, 2004, 2006, 2007, 2012.

[39] Licitra G, Hernandez TM, van Soest PJ (1996). Standardization of procedures for nitrogen fractionation of ruminant feeds. Anim Feed Sci Technol.

[40] Araba M, Dale NM (1990). Evaluation of protein solubility as an indicator of overprocessing soybean meal. Poult Sci.

[41] Association of Official Analytical Chemists International. Official methods of analysis. 17th ed. Gaithersburg, USA: AOAC Int.; 2000.

[42] Llames CR, Fontaine J (1994). Determination of amino acids in feeds: Collaborative Study. J Assoc Off Anal Chem.

[43] Commission Directive 98/64/EC (1998). Establishing community methods of analysis for the determination of amino acids, crude oils and fats, and olaquindox in feedingstuffs and amending Directive 71/393/EEC. Annex part A. Determination of amino acids. Off J Eur Union.

[44] Commission Directive 2000/45/EG (2000). Establishing community methods of analysis for the determination of vitamin A, vitamin E and tryptophan in feedingstuffs. Annex part C. Determination of tryptophan. Off J Eur Union.

[45] Fontaine J, Zimmer U, Moughan PJ, Rutherfurd SM (2007). Effect of heat damage in an autoclave on the reactive lysine contents of soy products and corn distillers dried grains with solubles. Use of the results to check on lysine damage in common qualities of these ingredients. J Agric Food Chem.

[46] International Organization for Standardization 9167-1:1992 (en). Rapeseed —Determination of glucosinolates content - Part 1: Method using high-performance liquid chromatography. Geneva, Switzerland: ISO; 1992.

[47] SAS. SAS User's Guide: Statistics. Inst. Inc., Cary, NC; 2008.

[48] National Research Council (NRC) (2012). Nutrient Requirements of Swine.

[49] Grala W, Verstegen MWA, Jansman AJM, Huisman J, van Leeusen P (1998). Ileal apparent protein and amino acid digestibilities and endogenous nitrogen losses in pigs fed soybean and rapeseed products. J Anim Sci.

[50] Pastuszewska B, Jabłecki G, Buraczewska L, Dakowski P, Taciak M, Matyjek R, Ochtabińska A (2003). The protein value of differently processed rapeseed solvent meal and cake assessed by in vitro methods and in tests with rats. Anim Feed Sci Technol.

[51] Classen HL, Newkirk RW, Maenz DD (2004). Effects of conventional and novel processing on the feed value of canola meal for poultry. Proc Aust Poult Sci Symp.

[52] Bjergegaard C, Eggum BO, Jensen SK, Sørensen H (1991). Dietary fibres in oilseed rape: Physiological and antinutritional effects in rats of isolated IDF and SDF added to a standard diet. J Anim Physiol Anim Nutr.

[53] van Soest PJ, Mason VC (1991). The influence of the Maillard reaction upon the nutritive value of fibrous feeds. Anim Feed Sci Technol.

[54] Ramírez-Jiménez A, García-Villanova B, Guerra-Hernández E (2001). Effect of toasting time on the browning of sliced bread. J Sci Food Agric.

[55] Pahm AA, Pedersen C, Stein HH (2008). Application of the reactive lysine procedure to estimate lysine digestibility in distillers dried grains with solubles fed to growing pigs. J Agric Food Chem.

[56] Finot PA, Magnenat E (1981). Metabolic transit of early and advanced Maillard products. Prog Food Nutr Sci.

[57] Hurrell RF, Carpenter KJ (1981). The estimation of available lysine in foodstuffs after Maillard reactions. Prog Food Nutr Sci.

[58] van Barneveld RJ, Batterham ES, Norton BW (1994). The effect of heat on amino acids for growing pigs. 1. A comparison of ileal and faecal digestibilities of amino acids in raw and heat-treated field peas (*Pisum sativum* cultivar Dundale). Br J Nutr.

[59] Gerrard JA (2002). Protein–protein crosslinking in food: Methods, consequences, applications. Trends Food Sci Technol.

[60] Pastuszewska B, Buraczewska L, Ochtabińska A, Buraczewski S (1998). Protein solubility as an indicator of overheating rapeseed oilmeal and cake. J Anim Feed Sci.

[61] Campbell LD, Slominski BA (1990). Extent of thermal decomposition of indole glucosinolates during the processing of canola seed. J Am Oil Chem Soc.

[62] Association Française de Zootechnie, Ajinomoto Eurolysine, Aventis Animal Nutrition, Institut National de la Recherche Agronomique / UMRVP, Institut Technique des Céréales et des Fourrages. Paris, France: AmiPig: Ileal standardized digestibility of amino acids in feedstuffs for pigs; 2000.

[63] Oginsky EL, Stein AE, Greer MA (1965). Myrosinase acitivity in bacteria as demonstrated by the conversion of progoitrin to goitrin. Proc Soc Exp Biol Med.

